# Achondroplasia with 47, xxy karyotype: a case report of the neonatal diagnosis of an extremely unusual association

**DOI:** 10.1186/1471-2431-12-88

**Published:** 2012-06-29

**Authors:** Purificación Ros-Pérez, Francisco J Regidor, Esmeralda Colino, Cristina Martínez-Payo, Eva Barroso, Karen E Heath

**Affiliations:** 1 Department of Pediatrics, Hospital Universitario Puerta de Hierro-Majadahonda, C/Manuel de Falla 1, Majadahonda, 28222, Madrid, Spain; 2 Department of Gynecology and Obstetrics, Hospital Universitario Puerta de Hierro-Majadahonda, Madrid, Spain; 3 Institute for Medical and Molecular Genetics (INGEMM), Hospital Universitario La Paz and Instituto para la Investigación de La Paz (IdiPAZ), and CIBERER, ISCIII, Madrid, Spain

**Keywords:** Klinefelter syndrome, Achondroplasia, Mutation, Karyotype, Prenatal diagnosis

## Abstract

**Background:**

The association of achondroplasia and Klinefelter syndrome is extremely rare. To date, five cases have been previously reported, all of them diagnosed beyond the postnatal period, and only one was molecularly characterized. We describe the first case of this unusual association diagnosed in the neonatal period, the clinical findings and the molecular studies undertaken.

**Case presentation:**

The boy was born at term with clinical and radiological features indicating the diagnosis of achondroplasia or hypochondroplasia combined with the prenatal karyotype of Klinefelter syndrome (47,XXY). Neonatal *FGFR3* mutation screening showed that the newborn was heterozygous for the classic achondroplasia G340R mutation. Microsatellite marker analysis showed that the sex chromosome aneuploidy had arisen from a non-disjunction error in paternal meiosis I, with a recombination event in the pseudoautosomal region 1 (PAR1).

**Conclusion:**

Specific mutation analysis is appropriate to confirm the clinical diagnosis of achondroplasia for appropriate diagnosis, prognosis, and genetic counseling, especially when the karyotype does not explain the abnormal prenatal sonographic findings. In the present case, a recombination event was observed in the PAR1 region, although recombinational events in paternally derived Klinefelter syndrome cases are much rarer than expected.

## Background

The association of achondroplasia and Klinefelter syndrome is extremely rare. To our knowledge, only five cases have been reported previously [1-5], all well beyond the immediate postnatal period, and mutation analysis was performed in only one case [5]. In all cases the achondroplasia FGFR3 gene defect dominated the clinical picture, even prenatally, and the Klinefelter syndrome is not able to counter the achondroplasia’s effects on height. Klinefelter syndrome is readily diagnosed by karyotype analysis, but the diagnostic confirmation of the skeletal dysplasia requires a specific mutation analysis, necessary for genetic counseling and prognosis, and to determine perinatal lethality.

We clinically and molecularly describe here the first case of this extremely unusual association that was diagnosed in the neonatal period, and emphasize the importance of performing specific genetic studies when there are prenatal ultrasound findings suggestive of skeletal dysplasia. It is noteworthy that in our case a recombination event was observed in the PAR1, although a highly significant reduction in recombination has been observed in paternally derived Klinefelter syndrome cases.

## Case presentation

The boy was born at term (41 weeks according to gestational age) after an uneventful pregnancy. Birth weight was 3000 g (-0.77 standard deviations, SD), birth length 47 cm (-1.76 SD) and head circumference 36 cm (+ 0.6 SD), according to Spanish neonatal standards [6,7]. He was the second child of healthy parents of normal height (mother -0.6 SD, father - 1 SD). The mother was 37 years old and the father was 43 years old at the time of conception. There was no history of consanguinity. One brother was healthy. During pregnancy, a routine sonography was carried out at a different institution at 27 + 2 weeks and the fetus was detected to have abnormal head morphology with small body and limbs with no other significant abnormalities. Prenatal analysis was undertaken, revealing a 47,XXY karyotype, corresponding to Klinefelter syndrome. Genetic counseling was given accordingly and no further studies were undertaken at that time.

**Figure 1:**
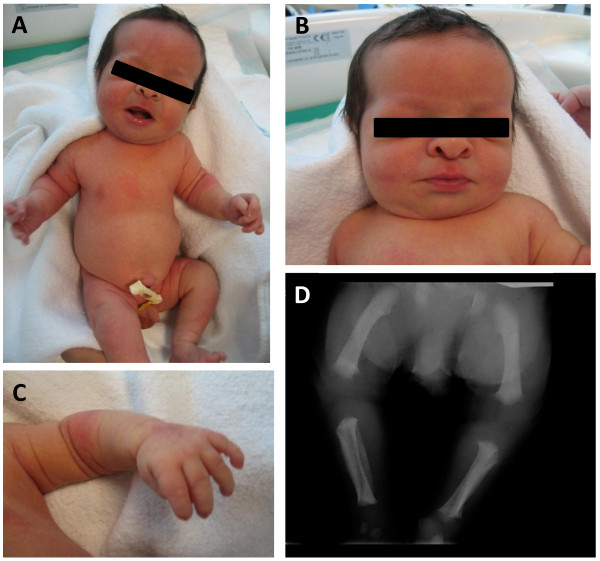
**Clinical and radiological findings. A**. Patients general appearance with rhizomelic limb shortening. **B**. A large head with protruding forehead, mid facial hypoplasia, depressed nasal bridge, hypoplasic chin, short neck. **C**. Short trident hands. **D**. Radiograph showing bowing and shortness of legs with metaphyseal flaring.

On physical examination at birth, the newborn had rhizomelic shortening, a large head with protruding forehead, mid-facial hypoplasia, depressed nasal bridge, hypoplastic chin, low set ears, and short neck (Figure 1A). The abdomen was protruding and there was evidence of decreasing anterior-posterior chest diameter, and lumbar lordosis. Signs of trident hand were present (Figure 1B). The external genitalia were unremarkable, with testes present in the scrotum that appeared normal (Figure 1C).

Radiological studies revealed decreased interpedicular distances, anterior beaking and posterior scalloping of the lumbar vertebrae (Figure 1D). The sacro-coccyx was curved posteriorly with small square iliac wing and the horizontal acetabulum roof had champagne glass appearance. The neonate had bilateral short humeri, radii and ulnae with metaphyseal flaring, as well as a short tibia and fibula with metaphyseal flaring, mildly increased fibular length and a generalized brachydactylia with bullet shaped proximal and middle phalanges. The clinical and radiological features indicated a diagnosis of achondroplasia or hypochondroplasia combined with the prenatal karyotype of Klinefelter syndrome. Hormonal analysis was also undertaken at day 3. LH (< 0.1 UI/L), FSH (< 0.3 UI/L) and testosterone (89.5 ng/dl) levels were within the normal range for age.

Mutation screening of exons 8 and 11 of *FGFR3* (NM_000142) was undertaken by PCR and direct sequencing. Mutations in these two exons account for >98% of achondroplasia and >70% of hypochondroplasia cases, including the most frequently observed mutations in achondroplasia (p.G380R) and hypochondroplasia (p.N540K). The patient was shown to be heterozygous for the classical c.1138 G > A mutation which results in a Glycine to Arginine substitution at position 380 (p.G380R) within the transmembrane domain of the mature protein.

**Figure 2:**
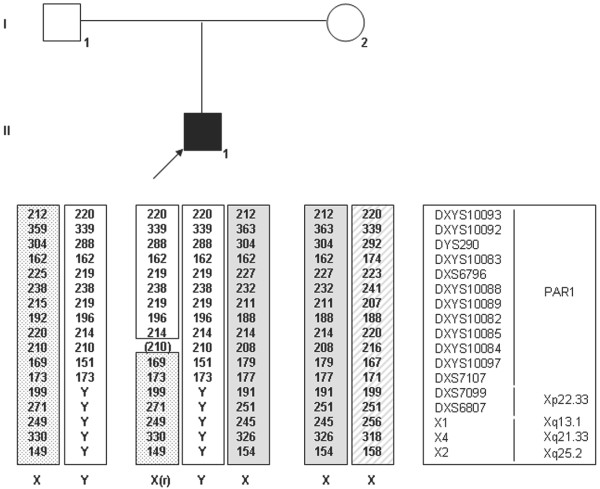
**Analysis of PAR1 and X-chromosome specific microsatellite markers in the studied family.** The markers show that the extra X chromosome is of paternal origin. A recombination is observed in PAR1 between markers DXYS10085 and DXYS10087. Marker DXYS10084 was not informative. X1, *X*2 and X4 are X-chromosome markers included in the Devyser Compact kit.

The 47,XXY karyotype was confirmed postnatally in 30 metaphases. Analysis of microsatellite markers localized along the X chromosome, including the pseudoautosomal region 1 (PAR1), was carried out as previously described [8,9] and in accordance to the kit (Devyser Compact). The aneuploidy was shown to be of paternal origin with a recombination event present between markers DXYS10082 and DXYS10097 (Figure 2). 

At the age of one year, the patient presented typical achondroplastic body proportions, normal neurological development except for axial hypotonia, and the height, weight and head circumference were -4.9 SD and -3.8 SD and -0.34 SD respectively, according to the Spanish population [6,7]. The patient developed a severe kyphoscoliotic deformity affecting the lower thoracic and upper lumbar spine.

## Discussion

To our knowledge, this is the first report of the extremely unusual association of achondroplasia and Klinefelter syndrome that was diagnosed in the neonatal period. Only five cases have been previously reported, including a 28 month old boy [4], one child [1], one teenager [5], and two adults [2,3]. The incidence of Klinefelter syndrome and achondroplasia occurring together is estimated at 1 in 25 million births using recent data for the respective disorders [10,11].

In previuously reported cases (1-5) the initial diagnosis was achondroplasia, based on the clinical findings of facial appearance, severe short stature and body proportions. Similarly, in our case, height was dominated by the effect of the FGFR3 mutation rather than by the XXY aneuploidy.

Based on the karyotype analysis ordered because of abnormal sonographic findings, our case was initially diagnosed prenatally in a different institution of non-mosaic Klinefelter syndrome. At birth, achondroplasia was clinically suspected and mutation analysis confirmed the suspicion. Our case is of great clinical interest, as it documents the interest of mutation screening when sonography during pregnancy reveals findings suggestive of a skeletal dysplasia, especially when the karyotype does not explain the abnormal sonographic findings.

The p.G380R substitution in the transmembrane domain of the FGFR3 protein, as identified in this case, is present in >98% of achondroplasia patients, and may either have arisen sporadically or be due to germline mosaicism. More than 90% of achondroplasia cases are sporadic and there is an association of increased incidence with advancing paternal age at the time of conception of affected individuals, as in this case, suggesting that *de novo* mutations are of paternal origin [10]. Recently, Almeida et al. [12] emphasized the clinical heterogeneity of skeletal dysplasias (achondroplasia and hypochondroplasia) associated with *FGFR3* mutations, and the importance of undertaking mutation analysis to confirm the clinical diagnosis and to establish an appropriate prognosis and genetic counseling [12].

Analysis of PAR1 microsatellite markers revealed that the extra sex chromosome was of paternal origin, arising from the fusion of an XY spermatozoon and an X ovum. The frequency of XY sperm increases with paternal age [13], thus increasing the risk of fathering boys with Klinefelter syndrome. Interestingly, in our case a recombination event was observed in PAR1, although a highly significant reduction in recombination has been observed in paternally derived cases of Klinefelter syndrome and in diploid 24, XY spermatozoa reviewed in [14].

Fertility prognosis in these cases is unclear. There is one report of preserved fertility in a non-mosaic Klinefelter patient with a mutation in *FGFR3* (5). The authors speculate that the classical FGFR3 mutation (p.G380R) found in this patient may stimulate spermatogonial proliferation and/or prevent apoptosis, giving germ cells a selective advantage. In our case the LH, FSH and testosterone levels were in the normal range at the third day of life. However, a more prolonged follow up is necessary to determine the function of the hypothalamic-pituitary-gonadal axis and the potential for fertility.

## Conclusions

In summary, mutation screening is appropriate when sonography during pregnancy reveals findings suggestive of a skeletal dysplasia, especially when the karyotype does not explain the abnormal sonographic findings. The presence of *FGFR3* mutations dominates the clinical picture and its identification is important not only for genetic counseling and recurrence risk, but also to determine perinatal lethality, the severity of the dysplasia and prognosis. The coordinated collaboration between gynecologists, pediatricians and geneticists is appropriate.

## Consent

Written informed consent was obtained from both parents for publication of this Case report and any accompanying images. A copy of the informed consent is available for review by the Series Editor of BMC Pediatrics.

## Ethical approval

The genetic analysis was approved by the local ethical committee, Hospital Universitario La Paz. All studies were in compliance with the Helsinki Declaration.

## Competing interests

The authors declare that there are no competing interests, either financial or non-financial, that could be perceived as prejudicing the impartiality of the research reported.

## Authors’ contributions

PRP conceived the report. PRP, FJR, EC and CMP carried out clinical examinations and diagnostic interpretations. EB and KEH: designed, performed and interpreted the genetic studies. PRP, FJR, EC, CMP and KEH wrote and revised the manuscript. All authors read and approved the final manuscript.

## Pre-publication history

The pre-publication history for this paper can be accessed here:

http://www.biomedcentral.com/1471-2431/12/88/prepub
